# Plasma vascular endothelial growth factor 165 in advanced non-small cell lung cancer

**DOI:** 10.3892/ol.2014.2016

**Published:** 2014-03-31

**Authors:** AHMED ABDALLAH, MOHAMED BELAL, AHMED EL BASTAWISY, RABAB GAAFAR

**Affiliations:** 1 Faculty of Pharmacy, Al-Azhar University, Cairo 11884, Egypt; 2 Department of Medical Oncology, National Cancer Institute, Cairo University, Cairo 11796, Egypt

**Keywords:** vascular endothelial growth factor, non-small cell lung cancer

## Abstract

Currently, there is no serum marker that is routinely recommended for lung cancer. Therefore, the aim of the present study was to demonstrate that plasma vascular endothelial growth factor 165 (VEGF 165) may be a potential marker for advanced lung cancer. Lung cancer is the leading cause of cancer-related mortality worldwide, therefore, it is important to develop novel diagnostic techniques. The present prospective case control study included two groups of patients; a control group of healthy volunteers and a second group of patients with advanced non-small cell lung cancer (NSCLC). The plasma VEGF 165 levels were measured at baseline by ELISA prior to the first-line gemcitabine-cisplatin regimen. The high VEGF 165 expression level cut-off was >703 pg/ml, and the primary endpoint was used to compare the plasma VEGF 165 levels between the NSCLC patients and the control group subjects. The secondary endpoint was used to identify the correlations between high VEGF 165 levels and; clinical response (CR), progression-free survival (PFS) and overall survival (OS) in the advanced NSCLC patients. In total, patients with advanced NSCLC (n=35) were compared with a control group of age- and gender-matched healthy subjects (n=34). The follow-up period was between Oct 2009 and Oct 2012, with a median follow-up time of 10.5 months. The median plasma VEGF 165 level was 707 pg/ml in the NSCLC patients versus 48 pg/ml in the healthy control subjects (P<0.001). However, no significant correlation was found between the plasma VEGF 165 levels and CR (P<0.5), median PFS (P=1.00) or OS (P=0.70). Therefore, it was concluded that plasma VEGF 165 may serve as a potential diagnostic marker for advanced NSCLC.

## Introduction

Lung cancer is the leading cause of cancer-related mortality worldwide (1). In addition, the prognosis of lung cancer is poor and the disease is rarely curable, with an overall five-year survival rate of ~15% (2). Therefore, the development of novel diagnostic techniques, to identify the disease in the early stages and for the follow-up of its progression, is important for more effective treatment and improved prognosis.

Vascular endothelial growth factor (VEGF) is the most significant growth factor that controls angiogenesis in normal and tumor cells (3). VEGF has been identified as a heparin-binding angiogenic growth factor, which exhibits high specificity for endothelial cells. Subsequently, it was realized that permeability-inducing factor and endothelial cell growth factor are encoded by a single VEGF gene, and that several VEGF isoforms are produced from this gene by alternative splicing to form active disulfide-linked homodimers. The VEGF gene is located on human chromosome 6 (4) and alternative splicing of VEGF mRNA accounts for at least six different isoforms from a single gene; 121, 145, 165, 183, 189 and 206 (5).

The VEGF isoforms differ in their heparin-binding properties, membrane association and secretion; VEGF 121 and 165 are the only freely soluble isoforms as the other isoforms are predominantly bound to heparin in the extracellular matrix (6). *In vivo*, only the three secreted isoforms, VEGF 121, 145 and 165, induce angiogenesis, with VEGF 165 being the predominant isoform that is secreted by benign and malignant cells (7).

The leading cause of mortality worldwide is cancer (specifically lung cancer) and metastases from cancer are the major cause of mortality, with angiogenesis (the growth of novel blood vessel networks) being a critical metastatic event. VEGF is the most important growth factor in controlling angiogenesis, and VEGF 165 is the predominant isoform that is secreted by benign and malignant cells for angiogenesis. Therefore, the aim of the present study was to evaluate the diagnostic value of VEGF 165 in advanced non-small cell lung cancer (NSCLC), by comparing VEGF 165 expression levels in an NSCLC patient group with those of the control group subjects. In addition, the correlations between VEGF 165 expression levels and; clinical response (CR), progression-free survival (PFS) and overall survival (OS) were evaluated.

## Patients and methods

### Subjects

The present study was conducted on 69 adults (aged 39–77 years) who were classified into two groups; a control group and an NSCLC patient group.

The control group consisted of 34 healthy volunteers without any chronic or acute diseases, including respiratory problems, and who were not on regular medication. The patient group consisted of 35 NSCLC patients who had presented to the chest section of the Department of Medical Oncology, National Cancer Institute, Cairo University (Cairo, Egypt). The patients were randomly selected, but met the inclusion criteria of having a confirmed diagnosis of NSCLC at an advanced stage (III or IV). All patients were newly diagnosed and had not yet received chemotherapy or radiotherapy, or undergone surgical resection of the cancer.

### Ethical approval

The present study was conducted according to the Declaration of Helsinki and the guidelines for Good Clinical Practice and approval was obtained from the local ethics committee of Cairo University, National Cancer Institute (Cairo, Egypt). Written informed consent was obtained from all patients prior to commencing the study.

### Inclusion criteria

For inclusion in the present study, the NSCLC patients were required to have a histologically confirmed diagnosis of lung cancer at stage IIIB or IV, while the controls were healthy volunteers without evidence of acute or chronic illness. The participants were required to be aged ≥18 years. The NSCLC patients had an Eastern Cooperative Oncology Group (ECOG) performance status of ≤2 and a life expectancy of at least six months. In addition, patients were required to have adequate bone marrow function, (white blood cell count, ≥3.0×10^9^/l; absolute neutrophil count, ≥1.5×10^9^/l; platelet count, ≥100×10^9^/l; and hemoglobin level, ≥9 g/l), liver function (serum bilirubin levels of ≤1.5 times the upper normal limit, alanine aminotransferase (ALT) and aspartate aminotransferase (AST) levels of up to three times those of the normal values, and ALT and AST levels of up to five times those of the normal limits allowed in patients with known liver metastases) and kidney function (plasma creatinine level, ≤1.5 times those of the normal value). Patients were required to be compliant, of a healthy mental state and within a geographical proximity that allowed adequate follow-up. In addition, the participants were required to provide written informed consent prior to any study-specific procedure.

### Exclusion criteria

Patients who were pregnant or breastfeeding, with a currently active second malignancy or involved in a current clinical trial were excluded from the present study.

### Treatment plan

The NSCLC patient group received the following chemotherapy regimen: Gemcitabine (1,000 mg/m^2^) i.v. in 250 cc normal saline (NS) over 30 min on days one and eight; and cisplatin (80 mg/m^2^ per day) i.v. in 500 cc NS over 1 h with standard hydration on day one. The regimen was administered every three weeks for up to six cycles in responding patients and an evaluation was performed every six weeks.

### Study assessment

The pretreatment assessment included a complete medical history and physical examination. Further assessments were conducted within seven days prior to treatment, which included vital signs, performance status (ECOG) and a complete blood count (CBC) with differential and full biochemical panels. Liver and renal function tests were performed and repeated prior to each treatment course.

Radiological evaluations, including computerized tomography (CT) scans of the chest and upper abdomen, were performed, as well as additional radiological imaging, such as bone scans as required..

Tumor response was evaluated according to the Response Evaluation Criteria in Solid Tumors as follows: i) Complete response (CR), complete disappearance of all known disease determined by two observations not less than four weeks apart; ii) partial response (PR), ≥30% reduction of the product of the perpendicular diameters of all measurable lesions; iii) stable disease (SD), <30% reduction or <20% increase in tumor size; and iv) progressive disease (PD), increase of >20% in the product of the perpendicular diameters of all measurable lesions, or the appearance of new lesions.

### Post treatment evaluation

Medical history and physical examination, as well as a CBC and chemical tests, including serum glutamic pyruvic transaminase, serum glutamic oxaloacetic transaminase, creatinine, Na, K and Ca levels, were performed every three weeks, while CT scans of the chest and upper abdomen were conducted every six weeks. Other investigations were performed as required.

### Statistical analysis

Data management and analysis were performed using the Statistical Package for Social Sciences version 17 (SPSS, Inc., Chicago, IL, USA). Data are presented as means ± standard deviation (SD), or as the median and ranges. Comparisons between the two groups were performed using Student’s t-test (8). P-values are two-sided and P<0.05 was considered to indicate a statistically significant difference.

### Measurement of VEGF 165 by sample collection

In total, 5-ml venous blood samples were withdrawn by EDTA into K_2_-containing BD vacutainers (purple-capped; Becton-Dickinson, Franklin Lakes, NJ, USA) from the NSCLC patients and healthy control subjects. The samples were concentrated using a Jumbosep™ Centrifugal Device (Pall Corporation, Port Washington, NY, USA) at 4,300.8 × g for 15 min, and the plasma was separated and stored at <−20°C until analysis was performed. Additionally, ELISA was used to assess the VEGF 165 plasma levels.

### Determination of VEGF 165

The microtiter plate provided in the VEGF165 ELISA kit (Wuhan EIAab Science Co., Ltd., Wuhan, China) was precoated with an antibody specific to VEGF 165. The standards and samples were added to the appropriate microtiter plate wells with a human monoclonal biotin-conjugated polyclonal antibody preparation specific to VEGF 165. Next, avidin conjugated to horseradish peroxidase was added to each microplate well and incubated. A 3,3′,5,5′-tetramethylbenzidine substrate solution was subsequently added to each well, and only the wells that contained VEGF 165, biotin-conjugated antibody and enzyme conjugated avidin exhibited a change in color. The enzyme-substrate reaction was terminated by the addition of a sulfuric acid solution and the color change was measured using an Eppendorf BioSpectrometer^®^ (Hamburg, Germany) at a wavelength of ±450 nm. The concentration of VEGF 165 in the samples was determined by comparing the optical density (OD) of the samples with the standard curve.

### Materials and components

The materials and components are listed in Table I; all reagents were brought to room temperature prior to use. To prepare 750 ml of wash buffer, 30 ml of wash buffer concentrate was diluted into deionized or distilled water. In addition, the standard was reconstituted with 1.0 ml of sample diluent, which produced a 5,000-pg/ml stock solution. The standard was gently agitated (vrn-210, Gemmy Industrial Corporation, Taipei, Taiwan) for ~10 min prior to making serial dilutions. The undiluted standard served as the highest standard (5,000 pg/ml), while the sample diluent served as the zero standard (0 pg/ml). For detection reagents A and B, a dilution was performed to the working concentration using assay diluents A and B (1:100), respectively.

### Assay procedure

All reagents were brought to room temperature and thoroughly mixed by gentle swirling prior to pipetting to avoid foaming. The appropriate number of strips were reserved for one experiment and the extra strips were removed from the microtiter plate. All the reagents, working standards and samples were prepared as described above.

A total of 100 μl of standard, blank and sample solution was added to each well, which were covered with the plate sealer and incubated for 2 h at 37°C. The solutions were removed from each well and were not washed. Detection reagent A working solution (100 μl) was added to each well, which was covered with the plate sealer and incubated for 1 h at 37°C. The process of aspirating and washing each well was repeated three times for three washes. Each well was washed with wash buffer (~400 μl) using a squirt bottle or multichannel pipette. Following the last wash, any remaining wash buffer was removed by aspiration, and by inverting and blotting the plate using clean paper towels. Detection reagent B working solution (100 μl) was added to each well and covered with a new plate sealer, followed by incubation for 1 h at 37°C. The aspiration/wash process was repeated for a further five times as conducted previously. Substrate solution (90 μl) was added to each well, which was covered with a new plate sealer and incubated for 30 min at 37°C protected from light. This was followed by the addition of stop solution (50 μl) to each well. When the color change was not apparently uniform, the plate was gently agitated to ensure thorough mixing. The OD of each well was determined using a TECAN microplate reader (Tecan Group, Ltd., Männedorf, Switzerland) at 450 nm.

## Results

### Subjects

The present study compared 35 patients with NSCLC (who had presented to the Department of Medical Oncology, National Cancer Institute, Cairo University) with age- and gender-matched healthy subjects that served as a control group (n=34). The patients comprised 28 males (80%) and seven females (20%), with ages ranging between 39 and 77 years. The clinicopathological characteristics of the patients are shown in Table II.

### VEGF 165 plasma levels

**Figure 1:**
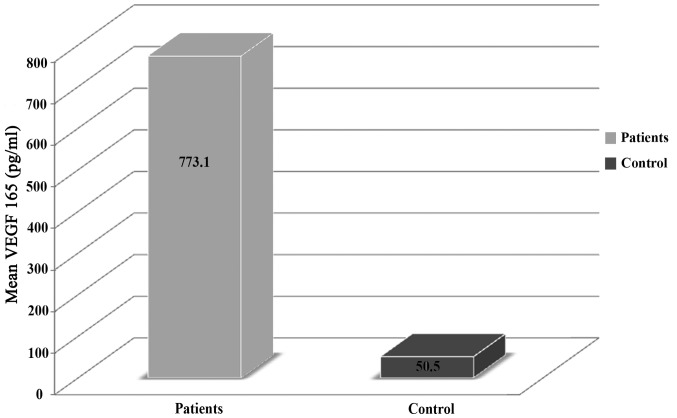
Comparison of the mean plasma levels of VEGF 165 in the non-small cell lung cancer patients and control group. VEGF, vascular endothelial growth factor.

The pretreatment plasma VEGF 165 levels of the NSCLC patients ranged between 452 and 2,058 pg/ml, with mean and median levels of 773.1 and 707. pg/ml, respectively. A statistically significant difference was identified in the VEGF 165 plasma levels between the NSCLC patients and the control group subjects (P<0.001; Table III). In addition, Fig. 1 shows the comparison of the mean plasma levels of VEGF 165 in the NSCLC patients and control group subjects.

### Correlation between plasma VEGF 165 levels, and age and gender

No significant correlations were identified between the VEGF 165 levels and age (P=0.45) or gender (P=0.70).

### Correlation between plasma VEGF 165 levels, and histopathological subtype

The patients with adenocarcinoma exhibited a mean plasma VEGF 165 level of 745.7±123.9 pg/ml (mean ± SD), while a mean level of 827.9±412.98 pg/ml (mean ± SD) was observed in patients with other pathological subtypes. However, this correlation was not identified to be statistically significant (P=0.41).

### Correlation between plasma VEGF 165 levels and stage

Patients were categorized as stage III or IV, and stage III patients exhibited a mean plasma VEGF 165 level of 793.54±397 pg/ml (mean ± SD), while patients categorized as stage IV exhibited a mean plasma VEGF 165 level of 794±285.1 pg/ml (mean ± SD). However, this difference was not identified to be statistically significant (P=0.17).

### Caorrelation between plasma VEGF 165 levels and CR

In total, 17 NSCLC patients achieved CR, PR and SD and notably, of these patients, eight (47%) exhibited low expression levels of VEGF 165 (≤703 pg/ml) and nine (53%) exhibited high expression levels of VEGF 165 (>703 pg/ml; P=0.50).

In addition, 18 NSCLC patients had PD, of which 10 (55%) exhibited low expression levels of VEGF 165 and eight (45%) exhibited high expression levels of VEGF 165 (P=0.50; Table IV).

### Correlation between plasma VEGF 165 levels, and PFS and OS

**Figure 2:**
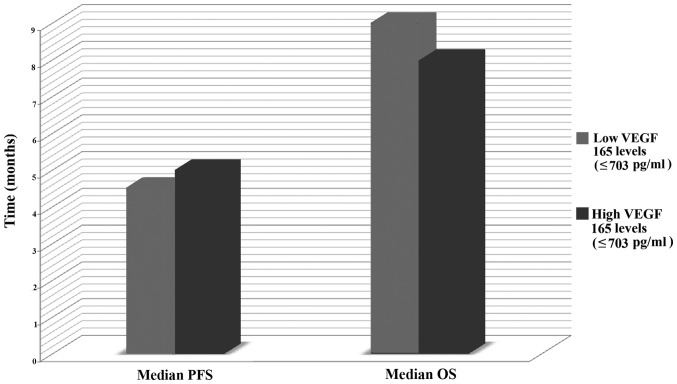
Correlation between plasma VEGF 165 levels and median PFS and OS. VEGF, vascular endothelial growth factor; PFS, progression-free survival; OS, overall survival.

**Figure 3:**
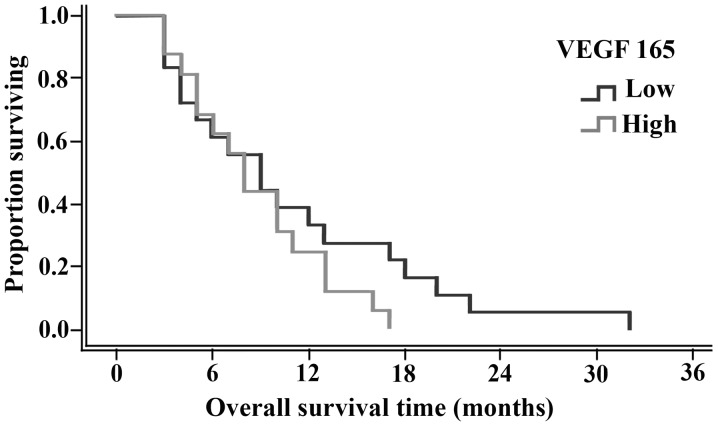
Overall survival curve according to the VEGF 165 plasma levels: Low VEGF 165, ≤703 pg/ml; and high VEGF 165, >703 pg/ml. VEGF, vascular endothelial growth factor.

**Figure 4:**
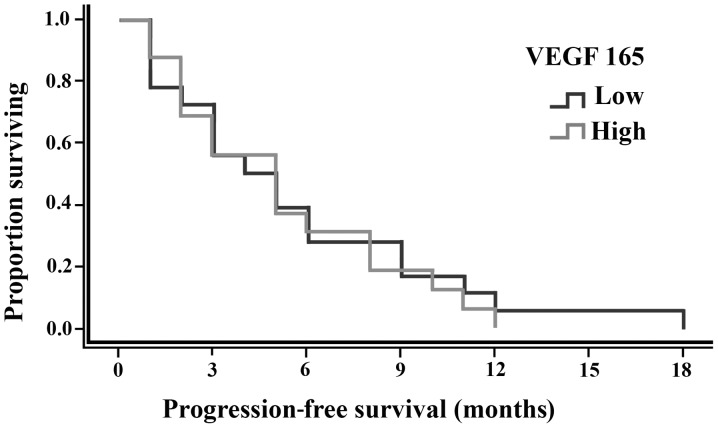
Progression-free survival curve according to the VEGF 165 plasma levels: Low VEGF 165, ≤703 pg/ml); and high VEGF 165, >703 pg/ml. VEGF, vascular endothelial growth factor.

The median plasma VEGF 165 level of the NSCLC patients was 707.0 pg/ml and ranged between 452 and 2,058 pg/ml (mean ± SD, 773.1±288.6 pg/ml). To evaluate the correlation between VEGF 165 levels, and PFS and OS the patients were divided into groups according to high (>703 pg/ml) or low (≤703 pg/ml) levels of VEGF 165 expression using the median value as a cut-off. Fig. 2 illustrates the correlations observed between plasma VEGF 165 levels and the median PFS and OS. The median PFS was 5 months (range, 1–18 months), while the median OS was 8.5 months (range, 3–32 months), however, no statistically significant differences were identified, as shown in Table V. In addition, OS and PFS curves are shown in Figs. 3 and 4, respectively.

## Discussion

Worldwide, cancer is the second most common cause of mortality following heart disease, with lung cancer being the leading cause of cancer-related mortality in males and the second leading cause in females; in 2008, there were an estimated 951,000 and 427,400 mortalities in males and females, respectively.

The prognosis of lung cancer is poor and the disease is rarely curable with an overall five-year survival rate of ~15% (2). The cure rates of lung cancer have remained relatively unaltered during the past 40 years. The high mortality rate is associated with the low cure rate (6–15%), which in turn is associated with the lack of adequate screening and early detection measures. Therefore, novel strategies for the screening and treatment of lung cancer disease are necessary for the improvement of patient outcome (9).

Previously, it has been shown that angiogenesis, a process where new blood vessels are formed by sprouting from a preexisting vasculature, is a relatively early event of carcinogenesis (3,10). Neovascularization is necessary for tumor growth of >2 mm^3^ and is essential for the adequate supply of oxygen and nutrients to the tissues (11).

VEGF is the most important growth factor controlling angiogenesis in normal and tumor cells, and its expression has been detected in a large variety of malignant human tumors (12).

It has been indicated that VEGF activates several critical gene products, which are involved in the VEGF-induced progression and metastasis of lung cancer (13,14). In addition, several studies have demonstrated that the mRNA expression (14–16) and serum levels of VEGF (15,17) are greater in patients with lung cancer when compared with those of healthy individuals. Other studies have shown the association between increased tumor or serum VEGF levels and poor survival (18–20), more advanced-stage lung cancer (13,18,20) and greater tumor size (21,22). Furthermore, VEGF serum level is considered to be a prognostic factor in patients with lung malignancies (20–24). It has also been reported that tumor angiogenesis, tumor growth and metastases are suppressed by the inhibition of VEGF signal transduction (25).

VEGF has numerous isoforms (≥12), however, Ferrara *et al* (26) reported that VEGF 121, 165 and 189 are the major isoforms secreted by the majority of cell types, with VEGF 165 the most abundant isoform found in normal and transformed cells (27). In addition, Dickinson *et al* (28) reported similar results, which identified that although nine alternatively spliced human VEGF isoforms have been described, three isoforms (VEGF 121, 165 and 189) predominate in the majority of human tissues and tumors. In particular, VEGF 165, and to a lesser extent VEGF 121, have been demonstrated as the predominant isoforms expressed in various human tumors, including astrocytomas, oligodendrogliomas and meningiomas (29–32).

Therefore, the aim of the present study was to evaluate the levels of VEGF 165 in the plasma of NSCLC patients and healthy control subjects, and to compare the expression levels with the patient survival rates.

To achieve this target, 69 subjects were enrolled in the present study and divided into two groups; a NSCLC patient group, including 35 patients with advanced stages of the disease at diagnosis prior to any type of treatment and a control group of 34 healthy subjects.

Hyodo *et al* (33) analyzed the stability of VEGF levels in plasma, in contrast to its instability in serum. The levels of serum VEGF in drawn blood samples were also found to increase during clot formation, which may be the result of VEGF release from platelets with slight contribution from leukocytes (34,35). Considering these results, the present study also measured the VEGF 165 levels in the plasma.

The majority of previous studies have investigated VEGF protein expression in lung carcinomas using immunohistochemical staining, while only a few studies have examined VEGF expression, rather than the different isoforms (such as VEGF 165), at the transcriptional level.

In the present study, a significant difference was identified in the VEGF 165 plasma levels between the NSCLC patients and the control group, with mean VEGF 165 plasma levels of 773.1 and 50.5 pg/ml for the NSCLC patients and control group, respectively (P<0.001). The expression levels ranged between 452 and 2,058 pg/ml in the patient group compared with between 29 and 86 pg/ml in the control group.

Few studies have analyzed the precise expression patterns of the four different VEGF isoform transcripts in the various normal and tumor tissues, and a limited number of studies have analyzed the translated isoforms. All studies identified concerning the transcriptional levels of VEGF 164 present results consistent with the results of the present study.

In a study by Tokunaga *et al* (36), the VEGF 189 or 165 mRNA isoform was found in 52 and 95% of colon cancers, respectively. In an additional study by Oshika *et al* (37), the VEGF 189 mRNA isoform was found in 90% of NSCLC samples, whereas all tumors expressed the VEGF 121 and 165 mRNA isoforms, and no expression of VEGF 206 mRNA was identified. These differences may result from different primer designs, varying polymerase chain reaction (PCR) efficiencies and the different patient populations that were used in the three studies.

The results of the current study are consistent with the results from a study by Zygalaki *et al* (38), who investigated the expression levels of the various VEGF splice variants in NSCLC and found the total expression of VEGF, VEGF 121 and 165 in all specimens, whereas the expression of VEGF 183 and 189 was only present in small amounts in certain samples. In addition, the total expression of VEGF, VEGF 121 and 165 mRNA was upregulated in cancerous tissues compared with that of the healthy tissues, whereas VEGF 183 and 189 expression tended to be higher in the healthy tissues.

In total, 40.7% of the patients included in the study by Timotheadou *et al* (39) were positive for the expression of VEGF 165 as determined by immunohistochemical and real-time quantified PCR analysis. Yuan *et al* (40) reported the expression of VEGF 121, 165 and 189 mRNA isoforms in all of their patients, but VEGF 206 mRNA isoform expression in only three patients.

The clinicopathological correlations with VEGF 165 expression identified in the current study for lung cancer were as follows: Lung cancer rarely occurred in patients prior to the age of 50 years and the incidence rates increased with age, peaking at ≥80 years for males and between 70 and 79 years for females (Globocan 2000; http://www.who.int/healthinfo/paper13.pdf). The median age of patients in the present case was 58 years. However, the results showed no significant correlation between VEGF 165 expression levels and age (<57.5 vs. >57.5 years; P=0.45) or gender (P=0.70). These results were consistent with the results reviewed in previous studies concerning the correlation between VEGF levels and age and gender (31–35,36–42). Although, one study revealed a correlation between VEGF 165 expression and age, which was consistent with the results of the present study (38).

Histologically, the three major subtypes of NSCLC are as follows: Adenocarcinoma, the most common subtype constituting for 54% of cases; squamous cell carcinoma, the second most common subtype accounting for 35% of NSCLC cases; and large cell carcinoma, the least common subtype, which accounts for ~11% of all NSCLC cases (44).

In the present study, 51.4% of patients had adenocarcinoma, 34.3% had squamous cell carcinoma and 14.3% had large cell carcinoma, however, no correlation was observed between the plasma levels of VEGF 165 and the different histological subtypes (P=0.40). Additionally, the majority of studies have shown no correlation between the serum levels of VEGF and the different histological types (13,15,45–51). In particular, Zygalaki *et al* (38) did not identify any significant differences between the expression of the various VEGF isoforms, including VEGF 165, and the different histopathological subtypes of NSCLC.

By contrast, other studies (16,22,52) demonstrated that patients with adenocarcinoma exhibit significantly higher VEGF expression levels than those with squamous cell carcinoma.

All of the patients included in the present study had advanced disease (stage III or IV); 12 patients (34%) with stage III disease and 23 (66%) with stage IV. However, no statistically significant correlation was identified between VEGF 165 expression and stage. In addition, Brattström *et al* (23), Takigawa *et al* (15) and Trapé *et al* (48) found similar results with regard to correlations between VEGF expression and stage, as well as Zygalaki *et al* (38) who also concluded the same results, with no correlation demonstrated between the expression of the investigated VEGF genes, including VEGF 165, and the different stages of the disease.

The present study investigated the correlation between the plasma VEGF 165 levels and PFS and OS. The patients were divided into high VEGF 165 (>703 pg/ml) or low VEGF 165 (≤703 pg/ml) expression groups, with the median value serving as a cut-off. The median plasma VEGF 165 level of the NSCLC patients was 707.0 pg/ml, ranging between 452 and 2,058 pg/ml (mean ± SD, 773.1±288.6 pg/ml). In addition, the median PFS was 5 months (range, 1–18 months), while the median OS was 8.5 months (range, 3–32 months). Overall, no statistically significant difference was identified in the median PFS between patients with high serum VEGF 165 levels (five months) and low serum VEGF 165 levels (4.5 months; P=1.00).

Additionally, in 2001, Yuan *et al* (40) found no statistically significant difference in OS and relapse time between patients with high or low tumor mRNA expression ratios for VEGF 121, 165 or 206.

The general association between the expression of VEGF with the angiogenic status and prognosis of the lung cancer has been controversial. In a study conducted by Brattström *et al* (40) in 1998, in which NSCLC patients were treated with thoracic irradiation with or without chemotherapy, an elevated serum VEGF level did not demonstrate any prognostic significance. Furthermore, serum VEGF levels have not been identified as significant prognostic factors in several studies (14,53–58). However, a number of other studies have established the involvement of VEGF in tumor tissue to be a poor prognostic factor in NSCLC (22,50,59–61).

The discrepancy in results in the present study may be due to a number of reasons, including sample size, which was relatively small, as well as discrepancies in disease stage as no stage I or II patients were included.

In the present study, no statistical significance was demonstrated between the groups of high and low levels of plasma VEGF 165, although, the two groups were considered to be of high levels. This was confirmed by comparing the plasma VEGF 165 levels of the NSCLC patients that ranged between 452 and 2,058 pg/ml, with the levels of the subjects in the control group, which ranged between 29 and 86 pg/ml.

In conclusion, NSCLC patients express much higher plasma levels of VEGF 165 than healthy subjects, which indicates the involvement of VEGF 165 in the angiogenesis and the pathogenesis of the disease (unless VEGF 165 is identified as having an additional role).

The VEGF 165 plasma levels in advanced stage (III and IV) NSCLC were not found to correlate with age, gender, stage or the histopathological subtype. In addition, the high and low plasma levels of VEGF 165 in advanced stage (III and IV) NSCLC did not correlate with the patient OS or PFS, although, a larger sample size of patients is required to confirm this result.

Future studies are required to assess the various VEGF isoforms involved in the different stages of lung cancer, and to correlate the expression levels of the VEGF isoforms with the angiogenesis and pathogenesis of lung cancer in an attempt to use them as prognostic factors or targets for novel treatments.

## Figures and Tables

**Table I:** Vascular endothelial growth factor 165 test materials and components.

Item	Quantity
Assay plate, n	1
Standard, n	2
Sample diluent	20 ml
Assay diluent A	10 ml
Assay diluent B	10 ml
Detection reagent A	120 μl
Detection reagent B	120 μl
Wash buffer (X25)	30 ml
Substrate	10 ml
Stop solution	10 ml
Plate sealer for 96 wells, n	5

**Table II:** Clinicopathological characteristics of the 35 non-small cell lung cancer patients included in the study.

Characteristic	Value
Subjects, n (%)	35 (100)
Gender, n (%)	
Female	7 (20)
Male	28 (80)
Age, years	
Range	39–77
Median	58
Pathological subtype, n (%)	
Adenocarcinoma	18 (51.4)
Squamous cell carcinoma	12 (34.3)
Large cell carcinoma	5 (14.3)
Stage, n (%)	
III	12 (34)
IV	23 (66)
Smoking history, n (%)	
Smoker	29 (83)
Non-smoker	6 (17)

**Table III:** Comparison of the plasma levels of VEGF 165 in the NSCLC patients and control group.

Plasma VEGF level	NSCLC cases (n=35)	Controls (n=34)
Mean, pg/ml	773.1	50.5
Standard deviation, σ	288.6	13.3
Minimum, pg/ml	452	29
Median, pg/ml	707	48
Maximum, pg/ml	2,058	86
P-value	<0.001^a^	

a P<0.05 vs. NSCLC cases.

VEGF, vascular endothelial growth factor; NSCLC, non-small cell lung cancer.

**Table IV:** Correlation between patient plasma VEGF 165 levels and clinical response.

CR, PR and SD			PD		
	
High VEGF 165, n	Low VEGF 165, n	P-value	High VEGF 165, n	Low VEGF 165, n	P-value
9	8	0.5	8	10	0.5

CR, complete remission; PR, partial remission; SD, stationary disease; PD, progressive disease; VEGF, vascular endothelial growth factor.

**Table V:** Correlation between patient plasma VEGF 165 expression levels, and patient PFS and OS.

VEGF 165 level	n	Median PFS, months	Median OS, months
Low (≤703 pg/ml)	18	4.5	9
High (>703 pg/ml)	17	9	8
P-value		1	0.7

VEGF, vascular endothelial growth factor; PFS, progression-free survival; OS, overall survival.
